# One Definition to Join Them All: The N-Spherical Solution for the EEG Lead Field

**DOI:** 10.3390/s23198136

**Published:** 2023-09-28

**Authors:** Ricardo Bruña, Giorgio Fuggetta, Ernesto Pereda

**Affiliations:** 1 Center for Cognitive and Computational Neuroscience, Universidad Complutense de Madrid (UCM), 28040 Madrid, Spain; eperdepa@ull.edu.es; 2 Department of Radiology, Rehabilitation and Physical Therapy, Universidad Complutense de Madrid (UCM), IdISSC, 28040 Madrid, Spain; 3 School of Psychology, University of Roehampton, London SW15 4JD, UK; giorgio.fuggetta@roehampton.ac.uk; 4 Department of Industrial Engineering, Institute of Neuroscience & Institute of Biomedical Technology, Universidad de La Laguna, 38200 Tenerife, Spain

**Keywords:** electroencephalography, source reconstruction, forward modeling

## Abstract

Albeit its simplicity, the concentric spheres head model is widely used in EEG. The reason behind this is its simple mathematical definition, which allows for the calculation of lead fields with negligible computational cost, for example, for iterative approaches. Nevertheless, the literature shows contradictory formulations for the electrical solution of this head model. In this work, we study several different definitions for the electrical lead field of a four concentric spheres conduction model, finding that their results are contradictory. A thorough exploration of the mathematics used to build these formulations, provided in the original works, allowed for the identification of errors in some of the formulae, which proved to be the reason for the discrepancies. Moreover, this mathematical review revealed the iterative nature of some of these formulations, which allowed us to develop a formulation to solve the lead field in a head model built from an arbitrary number of concentric, homogeneous, and isotropic spheres.

## 1. Introduction

The calculation of the distribution of electric potential due to the current in a conductor is not trivial. However, this calculation is necessary to reconstruct, for example, the cortical sources of activity of electroencephalography (EEG). Currently, the solution of this forward problem is usually calculated using numerical approaches, where the conductor (in this case, the head) is divided into a continuous series of elements of piecewise constant conductivity. However, for very simple cases where the geometry can be mathematically parametrized, it is possible to use a mathematical approach, which would give an “exact” solution for the problem. Since the head can be approximately modeled as a sphere, the widespread solution is that of a spherical conductor. While it is not precise for a realistic head, it is exact for spherical geometry.

The electrical lead field in the surface of a homogeneous sphere was mathematically solved by Fran in 1952 [1], and the solution for a multilayer concentric set of spheres was proposed by Arthur and Geselowitz in 1970 [2] and simplified by Cuffin and Cohen in 1979 [3]. The definition of the geometry-defined constants in the solution was not precise, and it was later improved by Lütkenhöner [4] and included in the FieldTrip toolbox [5]. In parallel, Yao and colleagues [6,7] and Nunez and Srinivasan [8] developed their own approaches. However, these three approaches seem, at first, incompatible. In addition, Lütkenhöner’s work is not publicly available, and Nunez and Srinivasan’s solution contained several errors, later corrected by Næss [9]. Right now, it is not clear if all these solutions define the same phenomena or if some of them are erroneous.

Although the increase in computational power allows for complex head models for solving the EEG forward problem, the use of computationally simple spherical solutions continues to be broadly extended [10,11]. For example, the spherical solution is still prevalent in cases where the accuracy of the forward solution is not essential [12] or as part of a two-step iterative procedure, where the simplified geometry is used in a first approach and a more realistic one is used in the last steps. Moreover, the existence of accurate analytical solutions is of paramount importance for the evaluation of the accuracy of numerical approaches. As these solutions are naïve to the parametrization of the geometry, their error will be similar in simple and realistic cases, and the concentric spheres model has been broadly used as testing ground [13,14,15,16].

In this work, we go over the three most common formulae for solving the lead field in a concentric spherical head model [3,6,8] and compare the different solutions. In addition, we create an alternative formulation that explodes the iterative nature of the previous solutions and allows for an arbitrary number of concentric spheres. Then, we evaluate the validity of this approach, comparing the results with implementations based in the previous literature. Finally, we provide an implementation of the algorithm to be freely used by the reader.

## 2. Mathematical Development

For a point-like current (i.e., an elemental dipole) P, in an infinite medium of electrical conductivity σ, located in the *Z*-axis and oriented radially, the potential in any point of space $\vec{r}$ is given by the solution to Laplace’s equation, which in spherical coordinates can be written as [2]

(1) $$\Phi_{\infty }\left(\vec{r}\right)=\frac{P}{4\pi \sigma }\sum_{l=1}^{\infty }\frac{{r_{0}}^{l}}{r^{l+1}}P_{l}\left(\cos\theta \right)$$

where $r_{0}$ is the distance from the dipole to the origin, r and θ are the radial and azimuthal coordinates of point $\vec{r}$, and $P_{l}$ is the Legendre polynomial of degree l. As the orientation of the dipole is radial (i.e., with the *Z*-axis), the potential distribution is rotationally symmetric, and the result is independent of φ.

When the medium is replaced with a set of s concentric spheres, of piecewise constant conductivity $\sigma_{s}$ and surrounded by vacuum (i.e., no conductivity), the solution for the potential is [3]

(2) $$\Phi^{s}\left(\vec{r}\right)=\frac{P}{4\pi \sigma_{s}}\sum_{l=1}^{\infty }\left[A_{l}^{s}\cdot r^{l}+\frac{B_{l}^{s}}{r^{l+1}}\right]\cdot l\cdot P_{l}\left(\cos\theta \right)$$

where s denotes the region, and $A_{l}^{s}$ and $B_{l}^{s}$ depend on the boundary conditions, particularly the position of the dipole, the radii of the spheres, and the conductivity of the compartments. As these constants specifically affect the terms of the spherical harmonics’ expansion given by $P_{l}$, they are sometimes referred as a spherical harmonics spectrum or spatial filters [6]. We can relate them with the boundaries between compartments: when the potential generated with a dipole P reaches a boundary between media, the behavior can be described as a double layer of charges distributed in the boundary, with one in the i-th medium and another in the i+1-th medium [17]. Then, this distribution of charges will themselves generate a perturbation in the electric field that will propagate both inwards and outwards [8], and $A_{l}^{s}$ and $B_{l}^{s}\;$ describe this propagation.

It is important to note that, in the following equations—and in the same fashion as in the definitions of $A_{l}^{s}$ and $B_{l}^{s}$ above—the subscript will represent the degree of the Legendre polynomial (l, in general), and the superscript will represent the boundary where the constant is calculated (s, in general).

Applying the boundary conditions (i.e., continuity of the current and potential across boundaries and no current leaving the outer sphere of the conductor), Cuffin and Cohen [3] provided a closed formula to calculate the potential on the external surface of a four-layer conductor. This formula proved later to be inexact and was corrected by Lütkenhöner [4]. Using a similar approach, Nunez and Srinivasan [8] (later corrected by Naess and colleagues [9]) developed a formula for the potential at any point in the conductor. The general formulation is similar to (2):

(3) $$\Phi^{s}\left(\vec{r}\right)=\frac{P}{4\pi \sigma_{1}{r_{0}}^{2}}\sum_{l=1}^{\infty }\left[A_{l}^{s}\cdot {\left(\frac{r}{R_{s}}\right)}^{l}+B_{l}^{s}\cdot {\left(\frac{R_{s}}{r}\right)}^{l+1}\right]\cdot l\cdot P_{l}\left(\cos\theta \right)$$

In their work, the authors provide formulae to calculate the terms $A_{l}^{s}$ and $B_{l}^{s}$ for a four-layer conductor (see the Appendix in [9] for details on the derivation of (4)–(14)):

(4) $$A_{l}^{1}=\frac{\frac{l+1}{l}\cdot \frac{\sigma_{1}}{\sigma_{2}}+Z_{l}}{\frac{\sigma_{1}}{\sigma_{2}}-Z_{l}}\cdot {\left(\frac{r_{0}}{R_{1}}\right)}^{l+1},$$

(5) $$B_{l}^{1}={\left(\frac{r_{0}}{R_{1}}\right)}^{l+1},$$

(6) $$A_{l}^{2}=\frac{A_{l}^{1}+{\left(\frac{r_{0}}{R_{1}}\right)}^{l+1}}{{\left(\frac{R_{1}}{R_{2}}\right)}^{l}+Y_{l}\cdot {\left(\frac{R_{2}}{R_{1}}\right)}^{l+1}},$$

(7) $$B_{l}^{2}=Y_{l}\cdot A_{l}^{2},$$

(8) $$A_{l}^{3}=\frac{A_{l}^{2}+B_{l}^{2}}{{\left(\frac{R_{2}}{R_{3}}\right)}^{l}+V_{l}\cdot {\left(\frac{R_{3}}{R_{2}}\right)}^{l+1}},$$

(9) $$B_{l}^{3}=V_{l}\cdot A_{l}^{3},$$

(10) $$A_{l}^{4}=\frac{A_{l}^{3}+B_{l}^{3}}{{\left(\frac{R_{3}}{R_{4}}\right)}^{l}+\frac{l}{l+1}\cdot {\left(\frac{R_{4}}{R_{3}}\right)}^{l+1}},$$

(11) $$B_{l}^{4}=\frac{l}{l+1}\cdot A_{l}^{4},$$

(12) $$V_{l}=\frac{\frac{l}{l+1}\cdot \frac{\sigma_{3}}{\sigma_{4}}-\frac{{\left(\frac{R_{3}}{R_{4}}\right)}^{l}-{\left(\frac{R_{4}}{R_{3}}\right)}^{l+1}}{\frac{l+1}{l}\cdot {\left(\frac{R_{3}}{R_{4}}\right)}^{l}+{\left(\frac{R_{4}}{R_{3}}\right)}^{l+1}}}{\frac{\sigma_{3}}{\sigma_{4}}+\frac{{\left(\frac{R_{3}}{R_{4}}\right)}^{l}-{\left(\frac{R_{4}}{R_{3}}\right)}^{l+1}}{\frac{l+1}{l}\cdot {\left(\frac{R_{3}}{R_{4}}\right)}^{l}+{\left(\frac{R_{4}}{R_{3}}\right)}^{l+1}}},$$

(13) $$Y_{l}=\frac{\frac{l}{l+1}\frac{\sigma_{2}}{\sigma_{3}}-\frac{\frac{l}{l+1}\cdot {\left(\frac{R_{2}}{R_{3}}\right)}^{l}-{V_{l}\cdot \left(\frac{R_{3}}{R_{2}}\right)}^{l+1}}{{\left(\frac{R_{2}}{R_{3}}\right)}^{l}+V_{l}\cdot {\left(\frac{R_{3}}{R_{2}}\right)}^{l+1}}}{\frac{\sigma_{2}}{\sigma_{3}}+\frac{\frac{l}{l+1}\cdot {\left(\frac{R_{2}}{R_{3}}\right)}^{l}-V_{l}\cdot {\left(\frac{R_{3}}{R_{2}}\right)}^{l+1}}{{\left(\frac{R_{2}}{R_{3}}\right)}^{l}+V_{l}\cdot {\left(\frac{R_{3}}{R_{2}}\right)}^{l+1}}},$$

(14) $$Z_{l}=\frac{{\left(\frac{R_{1}}{R_{2}}\right)}^{l}-\frac{l+1}{l}\cdot Y_{l}\cdot {\left(\frac{R_{2}}{R_{1}}\right)}^{l+1}}{{\left(\frac{R_{1}}{R_{2}}\right)}^{l}+Y_{l}\cdot {\left(\frac{R_{2}}{R_{1}}\right)}^{l+1}}.$$

Here, $R_{s}$ is the radius of sphere s, and $\sigma_{s}$ is the conductivity of the compartment bounded by it. The constants ${\dot{Z}}_{l}$, $Y_{l}$, and $V_{l}$, for their part, are proportionality constants relating the terms $A_{l}^{s}$ and $B_{l}^{s}$ for s greater than 1.

One of the main caveats of this formulation is that the position of the dipole is included in the terms. Thus, it must be calculated for each dipole position. However, we can rewrite the formulae in (4)–(11) extracting the dipole position as follows:

(15) $$A_{l}^{1}={\tilde{A}}_{l}^{1}\cdot {\left(\frac{r_{0}}{R_{1}}\right)}^{l+1},$$

(16) $${\tilde{A}}_{l}^{1}=\frac{\frac{l+1}{l}\cdot \frac{\sigma_{1}}{\sigma_{2}}+Z_{l}}{\frac{\sigma_{1}}{\sigma_{2}}-Z_{l}},$$

(17) $$A_{l}^{2}=\frac{{\tilde{A}}_{l}^{1}\cdot {\left(\frac{r_{0}}{R_{1}}\right)}^{l+1}+{\left(\frac{r_{0}}{R_{1}}\right)}^{l+1}}{{\left(\frac{R_{1}}{R_{2}}\right)}^{l}+Y_{l}\cdot {\left(\frac{R_{2}}{R_{1}}\right)}^{l+1}}=\frac{{\tilde{A}}_{l}^{1}+1}{{\left(\frac{R_{1}}{R_{2}}\right)}^{l}+Y_{l}\cdot {\left(\frac{R_{2}}{R_{1}}\right)}^{l+1}}\cdot {\left(\frac{r_{0}}{R_{1}}\right)}^{l+1}={\tilde{A}}_{l}^{2}\cdot {\left(\frac{r_{0}}{R_{1}}\right)}^{l+1},$$

etc. The same idea can be applied to $B_{l}^{s}$ to obtain the terms ${\tilde{B}}_{l}^{s}$. Now, we rewrite the potential in (3) as follows:

(18) $$\Phi^{S}(\overset{\to }{r})=\frac{P}{4\pi \sigma_{1}r_{0}^{2}}\sum_{l=1}^{\infty }{\left(\frac{r_{0}}{R_{1}}\right)}^{l+1}[{\overset{\sim }{A}}_{l}^{S}\cdot {\left(\frac{r}{R_{S}}\right)}^{l}+{\overset{\sim }{B}}_{l}^{S}\cdot {\left(\frac{R_{S}}{r}\right)}^{l+1}]\cdot l\cdot P_{l}(\cos\theta ).$$

We can further simplify the definitions for the constants by introducing a new constant, $U_{l}$, into (12)–(14):

(19) $$U_{l}=\frac{l}{l+1},$$

(20) $$V_{l}=\frac{U_{l}\cdot \frac{\sigma_{3}}{\sigma_{4}}-\frac{U_{l}\cdot {\left(\frac{R_{3}}{R_{4}}\right)}^{l}-U_{l}\cdot {\left(\frac{R_{4}}{R_{3}}\right)}^{l+1}}{{\left(\frac{R_{3}}{R_{4}}\right)}^{l}+U_{l}{\cdot \left(\frac{R_{4}}{R_{3}}\right)}^{l+1}}}{\frac{\sigma_{3}}{\sigma_{4}}+\frac{U_{l}\cdot {\left(\frac{R_{3}}{R_{4}}\right)}^{l}-U_{l}\cdot {\left(\frac{R_{4}}{R_{3}}\right)}^{l+1}}{{\left(\frac{R_{3}}{R_{4}}\right)}^{l}+U_{l}\cdot {\left(\frac{R_{4}}{R_{3}}\right)}^{l+1}}},$$

(21) $$Y_{l}=\frac{U_{l}\cdot \frac{\sigma_{2}}{\sigma_{3}}-\frac{U_{l}\cdot {\left(\frac{R_{2}}{R_{3}}\right)}^{l}-{V_{l}\cdot \left(\frac{R_{3}}{R_{2}}\right)}^{l+1}}{{\left(\frac{R_{2}}{R_{3}}\right)}^{l}+V_{l}\cdot {\left(\frac{R_{3}}{R_{2}}\right)}^{l+1}}}{\frac{\sigma_{2}}{\sigma_{3}}+\frac{U_{l}\cdot {\left(\frac{R_{2}}{R_{3}}\right)}^{l}-{V_{l}\cdot \left(\frac{R_{3}}{R_{2}}\right)}^{l+1}}{{\left(\frac{R_{2}}{R_{3}}\right)}^{l}+V_{l}\cdot {\left(\frac{R_{3}}{R_{2}}\right)}^{l+1}}},$$

(22) $$Z_{l}=\frac{1}{U_{l}}\cdot \frac{U_{l}\cdot {\left(\frac{R_{1}}{R_{2}}\right)}^{l}-Y_{l}\cdot {\left(\frac{R_{2}}{R_{1}}\right)}^{l+1}}{{\left(\frac{R_{1}}{R_{2}}\right)}^{l}+Y_{l}\cdot {\left(\frac{R_{2}}{R_{1}}\right)}^{l+1}}.$$

This formulation reveals the iterative nature of these constants. For the external compartment, the radius ($R_{5}$) is infinite, and the conductivity ($\sigma_{5}$) is zero. Therefore, the first constant, $U_{l}$, has the most straightforward definition. The rest of the constants depend on the previous one. However, the innermost constant, $Z_{l}$, does not follow this rule. We can also observe that, in (16), the definition of ${\tilde{A}}_{l}^{1}$ differs from that of the rest of ${\tilde{A}}_{l}^{i}$. To address this inconsistency, we can jointly rewrite $Z_{l}$ and ${\tilde{A}}_{l}^{1}$:

(23) $$Z_{l}=\frac{1}{U_{l}}\cdot \frac{U_{l}\cdot {\left(\frac{R_{1}}{R_{2}}\right)}^{l}-Y_{l}\cdot {\left(\frac{R_{2}}{R_{1}}\right)}^{l+1}}{{\left(\frac{R_{1}}{R_{2}}\right)}^{l}+Y_{l}{\cdot \left(\frac{R_{2}}{R_{1}}\right)}^{l+1}},$$

(24) $${\tilde{A}}_{l}^{1}=\frac{\frac{l+1}{l}\cdot \frac{\sigma_{1}}{\sigma_{2}}+Z_{l}}{\frac{\sigma_{1}}{\sigma_{2}}-Z_{l}}=\frac{\frac{\sigma_{1}}{\sigma_{2}}+U_{l}\cdot Z_{l}}{U_{l}\cdot \frac{\sigma_{1}}{\sigma_{2}}-U_{l}\cdot Z_{l}}=\frac{\frac{\sigma_{1}}{\sigma_{2}}+\frac{U_{l}\cdot {\left(\frac{R_{1}}{R_{2}}\right)}^{l}-Y_{l}\cdot {\left(\frac{R_{2}}{R_{1}}\right)}^{l+1}}{{\left(\frac{R_{1}}{R_{2}}\right)}^{l}+Y_{l}\cdot {\left(\frac{R_{2}}{R_{1}}\right)}^{l+1}}}{U_{l}\cdot \frac{\sigma_{1}}{\sigma_{2}}-\frac{U_{l}\cdot {\left(\frac{R_{1}}{R_{2}}\right)}^{l}-Y_{l}\cdot {\left(\frac{R_{2}}{R_{1}}\right)}^{l+1}}{{\left(\frac{R_{1}}{R_{2}}\right)}^{l}+Y_{l}\cdot {\left(\frac{R_{2}}{R_{1}}\right)}^{l+1}}}=\frac{1}{{\dot{Z}}_{l}},$$

(25) $${\dot{Z}}_{l}=\frac{U_{l}\cdot \frac{\sigma_{1}}{\sigma_{2}}-\frac{U_{l}\cdot {\left(\frac{R_{1}}{R_{2}}\right)}^{l}-Y_{l}\cdot {\left(\frac{R_{2}}{R_{1}}\right)}^{l+1}}{{\left(\frac{R_{1}}{R_{2}}\right)}^{l}+Y_{l}\cdot {\left(\frac{R_{2}}{R_{1}}\right)}^{l+1}}}{\frac{\sigma_{1}}{\sigma_{2}}+\frac{U_{l}\cdot {\left(\frac{R_{1}}{R_{2}}\right)}^{l}-Y_{l}\cdot {\left(\frac{R_{2}}{R_{1}}\right)}^{l+1}}{{\left(\frac{R_{1}}{R_{2}}\right)}^{l}+Y_{l}\cdot {\left(\frac{R_{2}}{R_{1}}\right)}^{l+1}}}.$$

We observe that the new constant ${\dot{Z}}_{l}$ follows the iterative form suggested in (19)–(21). This iterative nature can also be observed in the constants ${\tilde{A}}_{l}^{s}$ and ${\tilde{B}}_{l}^{s}$. To make it more evident, we rename the constants ${\dot{Z}}_{l}$, $Y_{l}$, $V_{l}$, and $Y_{l}$ as $X_{l}^{s}$ for s=1,…,4, respectively, and rewrite ${\tilde{A}}_{l}^{i}$:

(26) $${\tilde{A}}_{l}^{1}=\frac{1}{{\dot{Z}}_{l}}=\frac{1}{X_{l}^{1}},$$

(27) $${\tilde{A}}_{l}^{2}=\frac{{\tilde{A}}_{l}^{1}+1}{{\left(\frac{R_{1}}{R_{2}}\right)}^{l}+Y_{l}\cdot {\left(\frac{R_{2}}{R_{1}}\right)}^{l+1}}=\frac{{\tilde{A}}_{l}^{1}\cdot \left(1+{\dot{Z}}_{l}\right)}{{\left(\frac{R_{1}}{R_{2}}\right)}^{l}+Y_{l}\cdot {\left(\frac{R_{2}}{R_{1}}\right)}^{l+1}}=\frac{{\tilde{A}}_{l}^{1}\cdot \left(1+X_{l}^{1}\right)}{{\left(\frac{R_{1}}{R_{2}}\right)}^{l}+X_{l}^{2}\cdot {\left(\frac{R_{2}}{R_{1}}\right)}^{l+1}},$$

(28) $${\tilde{A}}_{l}^{3}=\frac{{\tilde{A}}_{l}^{2}+{\tilde{B}}_{l}^{2}}{{\left(\frac{R_{2}}{R_{3}}\right)}^{l}+V_{l}\cdot {\left(\frac{R_{3}}{R_{2}}\right)}^{l+1}}=\frac{{\tilde{A}}_{l}^{2}\cdot \left(1+Y_{l}\right)}{{\left(\frac{R_{2}}{R_{3}}\right)}^{l}+V_{l}\cdot {\left(\frac{R_{3}}{R_{2}}\right)}^{l+1}}=\frac{{\tilde{A}}_{l}^{2}\cdot \left(1+X_{l}^{2}\right)}{{\left(\frac{R_{2}}{R_{3}}\right)}^{l}+X_{l}^{3}\cdot {\left(\frac{R_{3}}{R_{2}}\right)}^{l+1}},$$

(29) $${\tilde{A}}_{l}^{4}=\frac{{\tilde{A}}_{l}^{3}+{\tilde{B}}_{l}^{3}}{{\left(\frac{R_{3}}{R_{4}}\right)}^{l}+U_{l}\cdot {\left(\frac{R_{4}}{R_{3}}\right)}^{l+1}}=\frac{{\tilde{A}}_{l}^{3}\cdot \left(1+V_{l}\right)}{{\left(\frac{R_{3}}{R_{4}}\right)}^{l}+U_{l}{\cdot \left(\frac{R_{4}}{R_{3}}\right)}^{l+1}}=\frac{{\tilde{A}}_{l}^{3}\cdot \left(1+X_{l}^{3}\right)}{{\left(\frac{R_{3}}{R_{4}}\right)}^{l}+X_{l}^{4}\cdot {\left(\frac{R_{4}}{R_{3}}\right)}^{l+1}}.$$

In this case, the innermost constant, ${\tilde{A}}_{l}^{1}$, is the simplest, and the other constants depend on the previous one.

In this form, if we take ${\left(\frac{r_{0}}{r}\right)}^{l+1}$ as a common factor, the constants can be seen to match the terms for the spatial filter $W_{l}^{s}$ in [7]

(30) $${\tilde{A}}_{l}^{s}\frac{r^{2l+1}}{{R_{1}}^{l+1}\cdot {R_{s}}^{l}}+{\tilde{B}}_{l}^{s}\cdot \frac{{R_{s}}^{l+1}}{{R_{1}}^{l+1}}=\frac{{\tilde{A}}_{l}^{s}}{{R_{1}}^{l+1}\cdot {R_{s}}^{l}}\left(r^{2l+1}+X_{l}^{s}{R_{s}}^{2l+1}\right)\equiv W_{l}^{s}.$$

Finally, taking advantage of the iterative nature of the definitions as mentioned above, we can write the constants for a conductor consisting of n concentric spheres as

(31) $$X_{l}^{n}=\frac{l}{l+1},$$

(32) $$X_{l}^{s}=\frac{X_{l}^{n}\cdot \frac{\sigma_{s}}{\sigma_{s+1}}-\frac{X_{l}^{n}\cdot {\left(\frac{R_{s}}{R_{s+1}}\right)}^{l}-X_{l}^{s+1}\cdot {\left(\frac{R_{s+1}}{R_{s}}\right)}^{l+1}}{{\left(\frac{R_{s}}{R_{s+1}}\right)}^{l}+X_{l}^{s+1}\cdot {\left(\frac{R_{s+1}}{R_{s}}\right)}^{l+1}}}{\frac{\sigma_{s}}{\sigma_{s+1}\;}+\frac{X_{l}^{n}\cdot {\left(\frac{R_{s}}{R_{s+1}}\right)}^{l}-X_{l}^{s+1}\cdot {\left(\frac{R_{s+1}}{R_{s}}\right)}^{l+1}}{{\left(\frac{R_{s}}{R_{s+1}}\right)}^{l}+X_{l}^{s+1}\cdot {\left(\frac{R_{s+1}}{R_{s}}\right)}^{l+1}}},$$

(33) $${\tilde{A}}_{l}^{1}=\frac{1}{X_{l}^{1}},$$

(34) $${\tilde{A}}_{l}^{s}=\frac{{\tilde{A}}_{l}^{s-1}\cdot \left(1+X_{l}^{s-1}\right)}{{\left(\frac{R_{s-1}}{R_{s}}\right)}^{l}+X_{l}^{s}\cdot {\left(\frac{R_{s}}{R_{s-1}}\right)}^{l+1}},$$

(35) $${\tilde{B}}_{l}^{s}=X_{l}^{s}\cdot {\tilde{A}}_{l}^{s}.$$

## 3. Evaluation of the Accuracy and Performance of the Proposed Implementation

In Section 2 of this work, we have developed an iterative formulation for the calculation of the potential at any point of a spherical conductor made from concentric spheres of homogeneous materials. In this section, we evaluate the accuracy of our formulation, compared with the classical formulations by Lutkenhoner, Yao, and Naess, together with the computational cost associated with each formulation. In this evaluation, we compare the different results using Lutkenhöner’s constant, as implemented in the FieldTrip toolbox [5], as the gold standard. The code for these comparisons is available in the GitHub repository of the work (https://github.com/rbruna/eeg-spheres/, accessed on 1 June 2023), where we provide implementations for the lambda constant using the mathematics described in the different works referenced in the main body of the manuscript.

**Figure 1:**
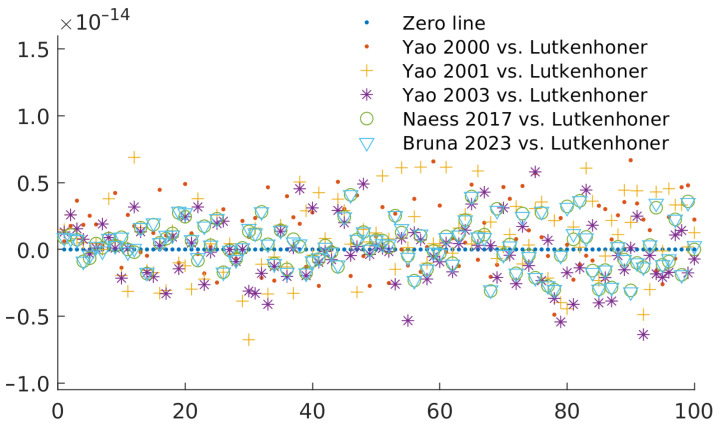
Relative error for each definition of the constant when using the default conductivity values (0.3333 S/m for the brain and the scalp and 0.3333/80 = 0.0042 S/m for the skull). The different values correspond to comparisons between Lutkenhöner [4] and Yao (2000) [6], Yao (2001) [12], Yao (2003) [7], and Næss (2017) [9], Bruna (2023): this research.

Figure 1 shows the error (compared to Lutkenhöner) as relative error (difference of values divided by the magnitude of the value) when using the classical values for the conductivities of the brain (1/3 S/m), the skull (1/(3 × 80) S/m), and the scalp (1/3 S/m). The error of all methods is similar and is inside the expected error for double-precision float point arithmetic.

**Figure 2:**
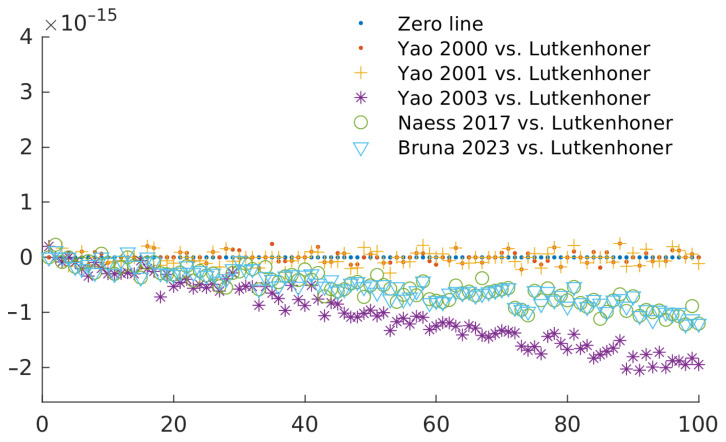
Relative error for each definition of the constant when using random conductivity values. The different values correspond to comparisons between Lutkenhöner [4] and Yao (2000) [6], Yao (2001) [12], Yao (2003) [7], and Næss (2017) [9], Bruna (2023): this research.

For a more general view, Figure 2 shows the relative error of each definition of the constant when using random (yet symmetrical) values for the conductivity. We can observe that the definitions of Yao in their 2000 and 2001 paper have the lowest relative error, while their definition (corrected as reported in the main body of the manuscript) in the 2003 paper had the largest one. The relative error in our proposed method is similar to that of Næss, which makes sense, as their definition is our main inspiration. Nevertheless, in all the cases, the relative error is inside the numerical error of the double-precision floating point operations.

Table 1 shows the times (averaged over 1 million executions) for the calculation of the constant for a three-sphere model. The implementation for Lutkenhöner’s definition is a closed formula and is by far the fastest but has the caveat that it can only be used to estimate the potential in the outer interface. The definitions by Yao are next, with the one from 2003 being the fastest (although the one with the larger error, see above). The implementation proposed here is approximately 30% faster than Næss, likely because their closed formula is defined for models with four spheres. In any case, the constant must be generated only once per head model, and the computation time for spherical harmonics of order 100 is less than a millisecond in all cases.

## 4. Discussion

In this work, we propose a reformulation of the solution for the potential in a conductor formed using a set of concentric spheres with piecewise constant conductivity. This reformulation reconciles the different definitions provided by Cuffin and Cohen [3], Nunez and Srinivasan [8], and Yao [7]. In doing so, we have also uncovered the iterative nature of the solution. Therefore, the formulation can be naturally extended to an arbitrary number of spheres.

The original algorithms used as the basis for this work are valid only for a fixed number of spheres, usually three [6] or four [3,7,8]. This limitation makes it necessary to use different algorithms for different models or to trick the algorithm by creating contiguous regions with the same conductivity, wasting precious CPU time. With the proposed formulation, the algorithm can be naturally extended to the required number of spheres without the need for optimization. In addition, we evaluated the performance and accuracy of the proposed formulation, showing that, while the solution is slightly slower that closed-form formulations, the accuracy is similar to those and close to the accuracy error for floating point arithmetic.

While the iterative nature of the solution allows for the calculation of the potential in a conductor with an arbitrary number of spheres, the practical applications of this feature are negligible. This is because, as the spherical volume conductor for EEG is an acceptable head model for fast calculation of the lead field in a real head, the geometry is not fit to the real head shape; therefore, an increase in the precision of the model will not tend toward an increased precision of the real volume conductor. However, this feature can be exploited in the evaluation of the accuracy of other, e.g., numerical, methods. For example, it is well known that classical BEM models are inaccurate in interfaces between media of different conductivity [18]. Some approaches have been developed to overcome this issue (as the isolated skull approach [19] or the symmetrical BEM methods [16]), but it is not clear how they will behave in more complex scenarios, for example, in the presence of low-conductivity pieces (i.e., fragments of skull). In this vein, the formulation proposed in this work could be of great use to evaluate more complex scenarios, with several high and low conductivities of different thickness layers interleaved.

Finally, in Appendix A, we provide an implementation of the algorithm, programmed in the MATLAB language and compatible with GNU Octave, to be used under the terms of GNU’s GPL version 3. This implementation can be compared with the classical formulations, and the code for such a comparison can be obtained from GitHub (https://github.com/rbruna/eeg-spheres/, accessed on 1 June 2023). Appendix B shows the results of these comparisons, both in relative error (compared to Lutkenhöner [4]) and in computation time.

In conclusion, the mathematical formulation provided in this work will help in understanding the behavior of the electrical currents in very simplified scenarios. Furthermore, as we provide a MATLAB code for the calculation of the potential, it can be readily used for anyone, without the need to generate their own implementation. And, as an unexpected extra, the feature of allowing models with an arbitrary number of spherical layers can help evaluate and improve the accuracy of numerical methods.

## Figures and Tables

**Table 1:** Average time for the execution, over 1 million runs, of each definition for a three-sphere model with order of the spherical harmonics up to 100.

Definition	Time (ms)
Proposed approach	0.198
Lutkenhöner [4]	0.064
Yao (2000) [6]	0.152
Yao (2001) [12]	0.177
Yao (2003) [7]	0.104
Næss (2017) [9]	0.268

## Data Availability

Not applicable.

## Appendix A. MATLAB Implementation of the Algorithm

Here, we present an implementation to get the gamma constant (Γ) in Cuffin and Cohen [3] using our iterative approach. As indicated in the main body of the manuscript, the definition of this constant in Cuffin and Cohen is incorrect, but the rest of the mathematics described in their work is sound.

The code provided below expects the following:

1. The concentric spheres are centered in the origin;
2. The radii of the spheres are provided as a vector with the name R;
3. The conductivities of the spheres are provided as a vector with the name S;
4. The spherical harmonics are to be evaluated until the order L.

The MATLAB implementation is as follows:

% Reserves memory for the X and A variables.

X = nan ( numel ( R ), order );

A_ = nan ( numel ( R ), order );

% Calculates the relative radii and conductivities.

Rr = R (:) ./R (:)′;

Sr = S (:) ./S (:)′;

% Creates the vector of orders from 1 to L.

Ls = 1: L;

% Gets the value of X for the outer sphere.

X ( end, : ) = Ls ./( Ls + 1 );

% Goes from the second-to-outer to the inner sphere.

for i = numel ( R ) - 1: -1: 1

 % Gets the first and previous values.

 Xn = X ( end, : );

 Xp = X ( i + 1, : );

 % Gets the repeated term.

 num = Xn .* ( Rr ( i, i + 1 ) .^ Ls ) - Xp .* ( Rr ( i + 1, i ) .^ ( Ls + 1 ) );

 den = ( Rr ( i, i + 1 ) .^ Ls ) + Xp .* ( Rr ( i + 1, i ) .^ ( Ls + 1 ) );

 term = num ./den;

 % Gets the value of X for the current sphere.

 num = Xn * Sr ( i, i + 1 ) - term;

 den = Sr ( i, i + 1 ) + term;

 X ( i, : ) = num ./den;

end

% Gets the value of A_ for the inner sphere.

A_ ( 1, : ) = 1 ./X ( 1, : );

% Goes from the second to the outer sphere.

for i = 2: numel ( R )

 % Gets the previous value of A_.

 A_p = A_ ( i - 1, : );

 % Gets the previous and current value of X.

 Xp = X ( i - 1, : );

 Xi = X ( i, : );

 % Gets the value of A_ for the current sphere.

 num = A_p .* ( 1 + Xp );

 den = ( Rr ( i - 1, i ) .^ Ls ) + Xi .* ( Rr ( i, i - 1 ) .^ ( Ls + 1 ) );

 A_ ( i, : ) = num ./den;

end

A4 = A_ ( end, : );

U = X ( end, : );

r1 = R ( 1 );

r4 = R ( end );

X4 = A4 ./r1 .^ ( Ls + 1 ) ./r4 .^ ( Ls );

% Calculates the spherical harmonics filter X at the outer layer.

X4 = X4 .* ( r4 .^ ( 2 * Ls + 1 ) + U .* r4 .^ ( 2 * Ls + 1 ) );

% Calculates gamma constant.

gamma = 1 ./( Ls .* X4 ./( 2 .* Ls + 1 ) .^ 4 );

## Appendix B. Potential Generated Using Dipoles with Arbitrary Orientation

In the main body of this work, we have presented the solution of the potential at an arbitrary point of a multi-layered spherical conductor. However, the final solution provided in (18) is only valid for radial dipoles located along the *Z*-axis. We opted to show the mathematical development in this scenario because, due to the rotational symmetry of the problem, the solution is also rotationally symmetric, and it shows no dependence with the polar coordinate φ. However, this definition is not complete, as it is not possible to describe any scenario with this equation. Here, we will describe the corresponding equations for a dipole located in the *Z*-axis but with an arbitrary orientation defined in cartesian coordinates.

Firstly, a radial dipole located over the *Z*-axis is oriented with the z coordinate. Therefore, (18) can be rewritten to describe the potential, $\Phi_{z}^{s}\left(\vec{r}\right)$, measured at an arbitrary point $\vec{r}$ of the s-th sphere and generated using a Z-oriented dipole of magnitude $P_{z}$.

(A1) $$\Phi_{z}^{s}\left(\vec{r}\right)=\frac{P_{z}}{4\pi \sigma_{1}{r_{0}}^{2}}\sum_{l=1}^{\infty }{\left(\frac{r_{0}}{R_{1}}\right)}^{l+1}\left[{\tilde{A}}_{l}^{s}\cdot {\left(\frac{r}{R_{s}}\right)}^{l}+{\tilde{B}}_{l}^{s}\cdot {\left(\frac{R_{s}}{r}\right)}^{l+1}\right]\cdot l\cdot P_{l}\left(\cos\theta \right).$$

When the dipole is tangential, the rotational symmetry disappears, and the resulting potential depends on φ. For the case of an X-oriented dipole of magnitude $P_{x}$, the potential $\Phi_{x}^{s}\left(\vec{r}\right)$ is described using [3,9]

(A2) $$\Phi_{x}^{s}\left(\vec{r}\right)=\frac{-P_{x}\cdot \cos\varphi }{4\pi \sigma_{1}{r_{0}}^{2}}\sum_{l=1}^{\infty }{\left(\frac{r_{0}}{R_{1}}\right)}^{l+1}\left[{\tilde{A}}_{l}^{s}\cdot {\left(\frac{r}{R_{s}}\right)}^{l}+{\tilde{B}}_{l}^{s}\cdot {\left(\frac{R_{s}}{r}\right)}^{l+1}\right]\cdot P_{l}^{1}\left(\cos\theta \right),$$

where $P_{l}^{1}$ is the associated Legendre polynomial of order 1 and degree l. Lastly, by rotating X over the *Z*-axis, we can obtain the potential $\Phi_{x}^{s}\left(\vec{r}\right)$ generated using a Y-oriented dipole of magnitude $P_{x}$:

(A3) $$\Phi_{y}^{s}\left(\vec{r}\right)=\frac{-P_{y}\cdot \sin\varphi }{4\pi \sigma_{1}{r_{0}}^{2}}\sum_{l=1}^{\infty }{\left(\frac{r_{0}}{R_{1}}\right)}^{l+1}\left[{\tilde{A}}_{l}^{s}\cdot {\left(\frac{r}{R_{s}}\right)}^{l}+{\tilde{B}}_{l}^{s}\cdot {\left(\frac{R_{s}}{r}\right)}^{l+1}\right]\cdot P_{l}^{1}\left(\cos\theta \right)$$

Nevertheless, the solutions for the potential provided in (A1)–(A3) are only valid for dipoles, either radial or tangential, located along the *Z*-axis. Other scenarios are possible, but the solution includes higher-order associated Legendre polynomials [2], and rotating the model is much more efficient.
